# Detection of attempted movement from the EEG during neuromuscular block: proof of principle study in awake volunteers

**DOI:** 10.1038/srep12815

**Published:** 2015-08-07

**Authors:** Yvonne Blokland, Loukianos Spyrou, Jos Lerou, Jo Mourisse, Gert Jan Scheffer, Geert-Jan van Geffen, Jason Farquhar, Jörgen Bruhn

**Affiliations:** 1 Radboud University Medical Centre, Department of Anaesthesiology, Pain and Palliative Medicine, P.O. Box 9101, 6500 HB Nijmegen, The Netherlands; 2 Radboud University Nijmegen, Donders Institute for Brain, Cognition and Behaviour, Montessorilaan 3, 6525 HR Nijmegen, The Netherlands

## Abstract

Brain-Computer Interfaces (BCIs) have the potential to detect intraoperative awareness during general anaesthesia. Traditionally, BCI research is aimed at establishing or improving communication and control for patients with permanent paralysis. Patients experiencing intraoperative awareness also lack the means to communicate after administration of a neuromuscular blocker, but may attempt to move. This study evaluates the principle of detecting attempted movements from the electroencephalogram (EEG) during local temporary neuromuscular blockade. EEG was obtained from four healthy volunteers making 3-second hand movements, both before and after local administration of rocuronium in one isolated forearm. Using offline classification analysis we investigated whether the attempted movements the participants made during paralysis could be distinguished from the periods when they did not move or attempt to move. Attempted movement trials were correctly identified in 81 (68–94)% (mean (95% CI)) and 84 (74–93)% of the cases using 30 and 9 EEG channels, respectively. Similar accuracies were obtained when training the classifier on the participants’ actual movements. These results provide proof of the principle that a BCI can detect movement attempts during neuromuscular blockade. Based on this, in the future a BCI may serve as a communication channel between a patient under general anaesthesia and the anaesthesiologist.

Detecting unintended awareness during surgery remains one of the biggest challenges in anaesthesia research and clinical practice. The incidence of awareness with postoperative explicit recall is currently 0.1–0.2%^1^,^2^. While these numbers are still a topic of debate^3^,^4^, the possible psychological sequelae for a patient are not. As the clinical signs of inadequate anaesthesia have proven to be unreliable, depth of anaesthesia monitors have been developed. Clinical estimates of depth of anaesthesia, such as the PRST–score (based on the observation of systolic blood Pressure, heart Rate, Sweating and Tears), are particularly masked by the effects of cardiovascular active medication in an ever-increasing proportion of the patients^5^.

Most commercially available depth of anaesthesia monitors are based on spontaneous frontal EEG activity^6^. With increasing anaesthetic drug concentrations characteristic changes in the spontaneous frontal EEG appear. After an initial increase in the beta band activity, the EEG slows down with a shift in the frequency spectrum to the theta and delta frequency bands. With further increasing anaesthetic drug concentrations burst suppression patterns appear with increasing phases of suppression until complete iso-electric activity. A simple way to track these changes with a single parameter from the power spectrum after Fourier Transformation is the median frequency, describing the frequency where 50% of the total power is above and 50% of the total power is below this frequency. Another parameter is the spectral edge frequency 95, describing the frequency where 95% of the total power is below this frequency. Both frequencies decrease with increasing anaesthetic drug concentrations. The disadvantages of these two approaches are “paradoxical” increases at lower anaesthetic drug concentrations caused by the beta increase and at high anaesthetic drug concentrations caused by the high frequency, high amplitude bursts during burst suppression^7^.

These issues have been solved by the commonly used Bispectral Index (BIS)^5^,^8^. The BIS is a multi-parameter index which incorporates a parameter from the frequency spectrum like the relative beta-ratio, parameters from the bispectrum and a burst suppression ratio. The BIS is the weighted sum of these features, trained and tested against data from patients who underwent different kinds of anaesthesia. The monitor’s output is a user-friendly number on a 0–100 scale (100 = awake, 0 = iso-electric). BIS values decrease with increasing anaesthetic drug concentrations. BIS values of 40–60 have been recommended as target during surgery under general anaesthesia. Use of the BIS monitor has been shown to reduce the incidence of intraoperative awareness^9^,^10^.

Nonetheless, the BIS monitor has several shortcomings. If using volatile anaesthetic drugs, like isoflurane or sevoflurane, the BIS is not superior to a regimen based solely on end-tidal drug concentration measurements regarding the incidence of intraoperative awareness^11^. The BIS also does not adequately reflect the effects of anaesthetic drugs like ketamine or nitrous oxide^12^. The standard frontal montage does not give insight in the deeper brain structures involved in consciousness and memory formation but the BIS is a “constructed abstract quantity that is not directly linked to any physiological parameter^13^”. Therefore it is not surprising that even the use of the bispectral index reduces but does not eliminate the incidence of intraoperative awareness. Furthermore a clear cut-off value —derived from the processed EEG— discriminating consciousness from unconsciousness is also missing. Whereas a range of BIS values of 40 to 60 is recommended during surgery, these values are quite arbitrarily chosen and the upper limit of 60 does not mean that every patient with a BIS value above 60 is conscious and every patient with a BIS value below 60 is unconscious^14^,^15^. Therefore, frontal cortical EEG measures a dose-dependent pharmacodynamic effect of anaesthetic drugs, but does not *per se* measure consciousness. The interested reader is referred to the literature for further details on monitors of anaesthetic depth^5^,^7^,^12^,^13^,^15^.

General anaesthesia involves the simultaneous administration of different components including a neuromuscular blocker for immobilization. Consequently, patients cannot communicate their awareness to the surgeon or anaesthetist even though they attempt to move^1^. We propose a novel monitor of intraoperative awareness based on detection of these movement attempts.

This paradigm follows the principles and techniques from the field of Brain-Computer Interfacing. A Brain-Computer Interface (BCI) measures a user’s brain signal, usually with electroencephalography, and translates this information to commands to drive a device or to enable communication. As it does not depend on overt behaviour such as speech or movement, a BCI can be beneficial for motor-impaired persons such as locked-in patients^16^. Patients under insufficient levels of anaesthetic drugs but nevertheless fully paralyzed by a neuromuscular blocker find themselves in a similar situation.

A well-known BCI paradigm is detection of imagined and attempted movement. Patterns of a power decrease in the mu and beta frequencies during motor tasks can be detected over the motor cortex to distinguish between left- and right hand motor tasks^17^, between movements of various body parts^18^,^19^, or simply between movement and lack of movement. The latter type of setup is often referred to as a ‘brain switch’, where one specific mental task is to be distinguished from a baseline state, and has been studied within the context of motor imagery on several occasions^20^,^21^.

A natural response for patients experiencing intraoperative awareness is trying to move^1^. Therefore, movement attempts are one of several possible indicators of awareness. Recent studies have shown that attempted movements could be distinguished from rest periods in patients with complete hand paralysis following stroke^22^ and patients with tetraplegia^23^. Similar results have been found even for patients in a locked-in state^24^. If attempted movements can also be detected during a drug-induced neuromuscular blockade, they could be used as input for a BCI-based monitor of intraoperative awareness.

We have previously discussed the requirements of such a monitor and shown that it is technically feasible, if based on detection of actual movements^25^. However, it is clinically important that the BCI is able to detect *attempted* movements as well. In the current proof-of-principle study we therefore obtained the electroencephalogram (EEG) from volunteers with a temporary paralysis of the forearm induced by administration of rocuronium, a commonly used neuromuscular blocker as a component of general anaesthesia. Using offline classification analysis we investigated whether the attempted movements the participants made during their paralysis could be distinguished from the periods when they did not execute or attempt movement.

## Methods

### The principles of a Brain-Computer Interface

A Brain-Computer Interface (BCI) is a system that, by means of a computer algorithm, interprets a user’s brain signals in order to convert them into some form of output that may aid the user in interacting with the environment.

EEG is the most commonly used brain measurement modality in BCI research because of its portability, relative affordability and high temporal resolution. It is recorded as the potential for current to pass between a recording electrode and a reference electrode. This potential is modulated when a cognitive task is being performed. Based on these modulations, the computer is required to distinguish between one task (such as movement) and another (such as absence of movement). By presenting the computer with examples of the brain signal produced during each task (from now on: ‘condition’) the algorithm learns the signal properties belonging to that condition.

When running a BCI two phases can be distinguished: the training phase and the test phase. In the training phase, prior to actual use of the BCI, a certain number of trials is measured for each condition. This information is used to train a classification algorithm (‘classifier’). In the test phase, for each novel trial the classifier makes a prediction of the intended condition based on what it has learned in the training phase. This prediction can be used real-time to generate a certain output. In our case, this could be an alarm or notification whenever attempted movement is predicted.

In an ‘offline’ study, such as the one presented here, no real-time output is produced. Instead, after collecting a sufficient number of trials, one part is used as the training data for the classification algorithm and the remainder for testing the algorithm. By determining the percentage of the test trials that has been predicted correctly, the classification accuracy is obtained (e.g. 80% when the classifier makes a correct decision for 8 out of 10 trials). This shows how reliably a certain mental task can be detected and thus how feasible the BCI paradigm is. In a two-class problem, with an equal number of trials for both conditions, the theoretical chance level performance is a classification accuracy of 50%. In other words, classification accuracies significantly higher than 50% mean that the EEG data contain useful information for a prediction of which condition a trial belongs to.

The interested reader is referred to van Gerven *et al.*^26^ for further details on the technology of BCI.

### Participants and consent

This study is registered in the EU Clinical Trials Register, identification number 2012-001777-86. All procedures were according to the Declaration of Helsinki and were approved by the local Medical Ethics Committee. Four right-handed healthy volunteers (aged 20–28, one female) participated in this study. None had any known neurological or motor impairments. Subject 1 had former BCI experience, while the other three were all naive to BCI. All participants gave written informed consent prior to the experiment. Measurements took place in an operating room at the Radboud University Medical Centre in Nijmegen, the Netherlands.

### Materials and procedures

The experimental paradigm consisted of two phases: one before, and one after administration of a neuromuscular blocker. In the first phase, participants were asked to perform four different types of movement: ‘actual movement’, ‘isometric movement’, ‘imagined movement’ and ‘no movement’. For subjects S1 and S2, the ‘actual movement’ task consisted of a repeated grasping movement of the right hand. For subjects S3 and S4, the actual movement performed was a repeated movement of the right-hand thumb towards the right-hand little finger. In the ‘imagined movement’ condition, participants had to perform kinesthetic motor imagery of the same movement as they performed in the ‘actual movement’ condition. During kinesthetic motor imagery, as compared to visual motor imagery, one imagines the feeling of performing movement rather than imagining seeing oneself perform that movement^27^. During ‘isometric movement’, the participants were instructed to perform the actual movement task, but reduce muscle movements as much as possible. This was used as an alternative to motor imagery as it was expected to more closely resemble the attempted movements^28^. In the ‘no movement’ condition subjects were instructed to keep still.

In the second phase, i.e. after drug administration, only two conditions remained: ‘actual movement’ and ‘no movement’. The instructions were identical to those in the first phase. Although participants were no longer able to physically perform the ‘actual movement’ task, they (mentally) performed the task as if they were. From now on, the ‘actual movement’ condition in the second phase will be referred to as ‘attempted movement’.

**Figure 1:**
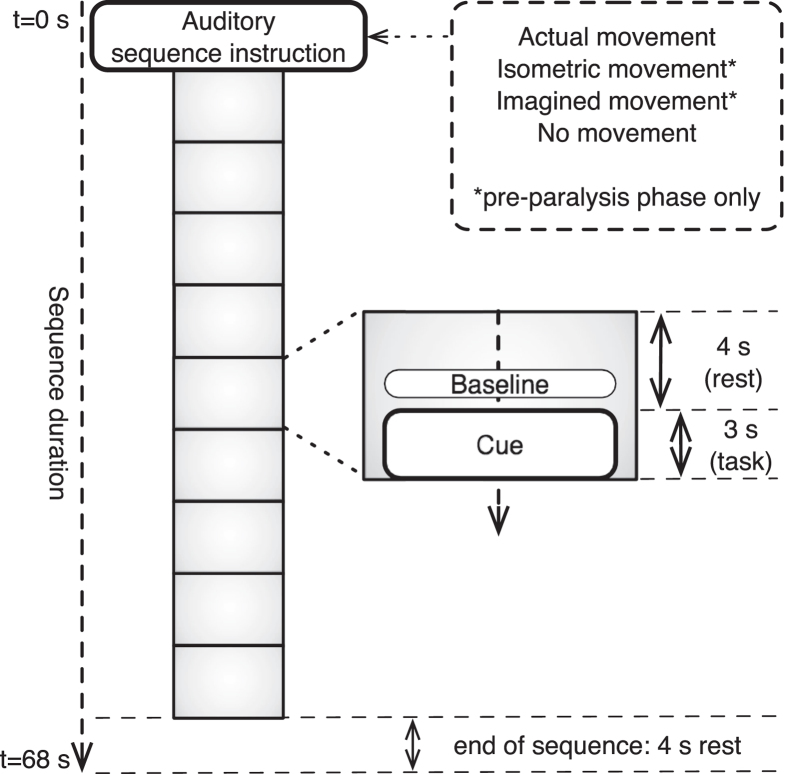
Visualization of experimental sequences.

Sequences of nine movement trials were presented to the subjects. Each trial consisted of an auditory 3-second cue, with a four-second silence interval between consecutive trials (Fig. 1). At the start of each sequence, an auditory instruction was given explaining the task for the upcoming trials. The participants had to perform the instructed task during the auditory cues, and rest during the silence intervals. Participants were asked to keep their eyes closed throughout the entire sequence. Between sequences participants could have a short rest, then start the next sequence by pressing a button. In total, 81 trials were collected for each of the four conditions in the first phase, divided over three experimental blocks, and 54 trials for both conditions in the second phase (single block). Within each block, presentation of the sequences was randomized. A short practice block to get the participants acquainted with the task preceded the actual measurements.

EEG was recorded with a 32-channel actiCAP system (Brain Products), with the channels positioned according to the international 10/20 system. Impedances were kept below 25 kΩ before starting the measurement. The signal was digitized with a sampling rate of 2500 Hz. Two electrodes were removed from the EEG cap and instead used to record the right forearm electromyogram (EMG) for S1 and S2, and the right-hand thumb EMG for S3 and S4. The experiment was programmed in and run on the BrainStream platform Version 1.0 (http://www.brainstream.nu), i.e. a Matlab (MathWorks Inc., MA, USA) toolbox especially developed for online BCI-experiments, using Psychtoolbox (http://psychtoolbox.org) for stimulus presentation.

### Drug administration

After phase one was finished, an iv-needle was inserted into a dorsal vein of the right hand to allow administration of the neuromuscular blocking agent rocuronium. An additional iv-needle was inserted into the left hand to enable quick administration of rescue medication if necessary. Then, after elevation of the right arm for two minutes, a tourniquet was applied to the right upper arm at 50 mmHg above the participant’s systolic blood pressure to separate the arm from the systemic circulation. Via the iv-needle 0.04 mg/kg rocuronium diluted with NaCl 0.9% to 20 ml was injected into the right arm. According to the protocol, a maximum of two extra doses of 0.01 mg/kg could be administered if the first dose was insufficient to establish full relaxation of the grasp muscles, bringing the maximum dose administered to 0.06 mg/kg. Subjects were asked to try to move every few minutes after drug administration. The level of relaxation was determined by visually inspecting both actual muscle movement and the EMG signals.

After phase 2, i.e. after the final experimental block had been completed, the tourniquet was deactivated. During the entire procedure heart rate, blood pressure and oxygen saturation (pulse oximetry) were monitored. Participants remained in the OR complex until their movement abilities were fully restored.

### Analyses

#### EMG

EMG recordings were used to determine the muscle output for each movement condition. EMG signals were re-referenced using a bipolar reference for the two channels and high-pass filtered at 10 Hz to reduce the effect of artifacts such as electrode drift. These signals were converted to power over time by taking the absolute magnitude of the analytic signal as found using a Hilbert transform, and then averaged for the period between 0.1 and 3.5 seconds (task onset is at 0). For each movement task and each subject the average power was calculated as a percentage of the average power during ‘actual movement’ for that subject.

#### Classification

The typical brain response to be seen during motor tasks is a power decrease in mu rhythm (8–12 Hz) and beta rhythm (18–25 Hz) activity in the sensorimotor cortex, with a short period of power increase in roughly the same frequencies after movement has stopped. These changes are commonly referred to as event-related desynchronization (ERD) and event-related synchronization (ERS)^29^. Thus, these were the main features the classifier was expected to use for its decisions.

To visually inspect the brain responses and ascertain the presence of ERD and ERS, for each subject and each movement condition a time-frequency plot was computed using a relative baseline from −1 to 6 seconds.

For the classification procedure, the data were first linearly detrended to minimize analysis artifacts due to large DC offsets. The surface Laplacian reference was calculated for each channel to reduce artifacts and increase signal strength, using Perrins spherical spline interpolation method^30^. In many SMR studies Common Spatial Patterns (CSP)^31^ is used for this purpose, however our initial comparison of both approaches showed no performance benefit when using CSP. Thus, we use the simpler unsupervised Laplacian reference here. Then, the power spectral density was computed for 8–24 Hz using Welch’s method^32^ with a resolution of 4 Hz and a Hanning taper applied to 50% overlapping windows (i.e. windows of 250 ms with overlap of 125 ms were used), using separate features for ERD (data obtained during movement, i.e. 0–3 s) and ERS (post-movement, i.e. 3.5–6 s). This subset of power spectral features (9 channels × 5 frequencies × 2 time ranges per epoch) was used to train a quadratically regularized linear logistic regression classifier (rLLR)^33^ to distinguish between each subjects specific pattern of spatial and spectral activation for the ‘actual movement’, ‘isometric movement’ and ‘imagined movement’ conditions as compared to the ‘no movement’ (first phase) condition, and between the activation for the ‘attempted movement’ condition as compared to the ‘no movement’ (second phase) condition. Regularization is used to limit the complexity of the classifier which prevents over-fitting in the high-dimensional input feature space. The optimal regularization strength (or equivalently classifier complexity) was found using a simple grid search with strengths of [.001 .01 .1 1 10 100] times the total data variance and selecting the strength which maximized validation set performance. Validation set performance was estimated using ten-fold cross-validation. So, for each condition the trials were split up into ten subsets (folds), with each fold used for testing once while the remaining nine folds were used for training the classifier.

Additionally, to more closely simulate a realistic clinical scenario including a pre-operative calibration phase, the classifier was trained on each movement condition from the first experimental part (as compared to the ‘no movement’ (first phase) condition) and then tested on the ‘attempted movement’ condition (as compared to ‘no movement’ (second phase)). As in this case the training and test sets consisted of different movement conditions by default, no cross-validation was needed for performance estimation.

Classification analyses were performed separately for two different channel sets. The first included all 30 EEG channels, the second consisted of a 9-channel subset located over the motor cortex (C3, C4, Cz, F3, F4, P3, P4, T7 and T8). The latter set is deemed more practical for clinical applications and has been found to lead to only a minimal reduction in classification performance^25^.

#### Statistics

For each condition, the 95% confidence interval (CI) for the mean classification accuracy was calculated using IBM SPSS Statistics version 20 (IBM Corp., NY, USA). As the number of ‘movement’ and ‘no movement’ trials was always equal, the theoretical chance level was 50%. Therefore, a 95% CI lower limit above 50% for a given condition means that the classifier performs better than chance (p = 0.05).

To determine significance at the individual subject level, we determined the 95% binomial CI for each classification accuracy (calculated in Matlab). Again, lower CI limits above 50% mean above-chance performance (p = 0.05).

## Results

The average time until full muscle relaxation was 18 minutes (range: 8–27). In one participant neuromuscular block was established within 8 minutes following a single dose of rocuronium (0.04 mg/kg). For three participants, an additional dose of 0.01 mg/kg was administered approximately 12 minutes after administration of the first dose. Of these three participants, one required a final dose of 0.01 mg/kg after another 10 minutes until full relaxation was established. The average duration of tourniquet activation was 32 minutes (range: 22–42). None of the participants reported tourniquet-induced pain or systemic effects of rocuronium after tourniquet deactivation. Within approximately one hour after tourniquet deactivation all participants left the OR facilities with their motor functioning restored to normal.

Subjects reported varying subjective experiences of the ‘attempted movement’ task. Although three out of four considered the task difficult, they all reported the sensation was to some extent similar to ‘actual movement’, more so than ‘imagined movement’. Only one subject reported having “the sensation of actually moving” during the ‘attempted movement’ task, although the EMG power during the neuromuscular block was only 0.8% of the power during actual movement. Subjective experiences of ‘isometric movement’ were very inconsistent, suggesting that despite extensive instructions and some practice trials, this task may have remained unclear or have been interpreted differently by each subject.

Analyses of the EMG measurements confirmed that muscle movement was blocked during the ‘attempted movement’ condition, with comparable power levels to the ‘no movement’ condition. The average EMG power per movement condition as a percentage of the power for ‘actual movement’ is shown for each participant in Table 1.

**Figure 2:**
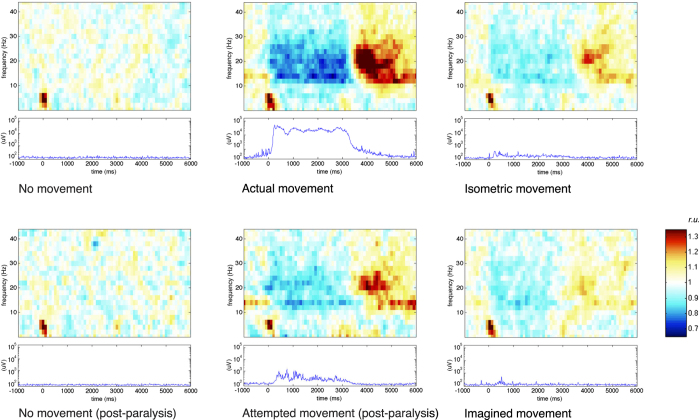
Grand average EEG time-frequency plots for channel C3 (top figures) and EMG power plots (bottom figures) per condition. For each movement condition the top plot shows EEG power over time per frequency in relative units (r.u.). These plots were computed to ascertain the presence of event-related desynchronization (ERD, blue) and -synchronization (ERS, red), the main features the classifier uses for its decisions. ‘Actual movement’, ‘Isometric movement’, ‘Attempted movement’ and ‘Imagined movement’ each show ERD during the movement task (t = 0–3 s), followed by ERS. A relative baseline over the entire trial was used, so that a value of 1 (white) represents average power, a value < 1 a power decrease or ERD and a value > 1 a power increase or ERS. The average EMG power over time is shown in the bottom plot. For the EMG plots a logarithmic scale is used on the y-axis.

Individual time-frequency plots of the EEG were examined for each subject and movement condition. ERD and ERS were visible in all subjects for ‘actual movement’, ‘attempted movement’, ‘isometric movement’ and ‘imagined movement’, with the exception of ‘imagined movement’ in subject 2. Figure 2 shows the grand average EMG power levels along with the time-frequency plot of the EEG for channel C3 for each movement condition. Individual plots can be found in the supplementary information.

Tables 2 and 3 show the classification results for both channel sets, along with their 95% CI’s. Overall classification accuracies for ‘imagined movement’ tended to be lower than for ‘actual movement’, ‘isometric movement’ and ‘attempted movement’. For the 30-channel set the average single trial classification accuracies were 84% for ‘actual movement’, 80% for ‘isometric movement’, 69% for ‘imagined movement’ and 81% for ‘attempted movement’. For the 9-channel set the rates were 87%, 78%, 71% and 84%, respectively. When training the classifier on ‘actual movement’ (first phase) and testing it on ‘attempted movement’ (second phase), single trial accuracies were 79% using the 30-channel set and 77% using the 9-channel set. For all conditions, the lower limit of the 95% CI was higher than 50%.

Table 2 shows that the lower limits of the 95% binomial CI’s for individual classification accuracies were above 50% for each movement condition, except for ‘imagined movement’ in one subject. So, classification performance was significantly better than chance for actual, attempted and isometric movement in every subject.

## Discussion

This study showed that attempted movements of the forearm paralyzed by a neuromuscular blocker can be detected from the EEG with a similar accuracy as movements that are truly executed. The average single trial classification accuracies were between 77% and 84%, irrespective of the number of EEG channels used, both when training on ‘attempted movement’ (same task) and on ‘actual movement’ (different task). Even the lowest of these mean classification accuracies with its associated 95% CI (77 (61–93)%), shows that the classifier performs significantly better than chance. Also on the level of individual classification accuracies the performance was found to be significant.

Therefore, like actual movements in healthy users and attempted movements in (partly) paralyzed patients, attempted movements blocked by a neuromuscular blocker may be used for communication. We propose the use of a Brain-Computer Interface converting detected movement attempts during surgery into an alert for the anaesthetist.

Compared to current EEG monitors, our proposed system has a more intuitive interpretation as it is based on a well-known neural response to motor tasks. Moreover, a BCI-monitor is based on the brain response of the individual patient, in contrast to current monitors that use a population-based probability of awareness at a given pharmacodynamic (surrogate) EEG effect.

Our finding that using actual movement rather than attempted movement for classifier training has little effect on the classification performance has important practical implications for the usability of the proposed monitor. For detection of *attempted* movement during general anaesthesia, pre-operative system calibration could be based on *actual* movements. However, it would be even better if a calibration session for each individual user would be made superfluous altogether by implementing a generic classifier^34^,^35^,^36^.

Another important result for the clinical usability of the proposed system was the confirmation of our previous findings^25^ that the average performance does not decrease when reducing the number of channels to nine. A system consisting of only nine EEG channels rather than a full standard cap with e.g. 32 channels would allow for a quick setup and is therefore clinically feasible.

This study used a cued design, meaning the user (patient) would have to perform the attempted movements during the indicated time periods. Ideally, the system would be able to detect spontaneous movement in an *asynchronous* setting. However, the time-lock now allows for easier incorporation of the post-movement beta-rebound (ERS). Converting the proposed paradigm to an asynchronous system is an important area for future research.

Although this study provides proof of our proposed concept, the paradigm requires further validation before it could be adopted in clinical settings. The most important limitation is that the participants were awake and no drugs other than the neuromuscular blocking agent were administered. As a first step towards building a BCI for use during general anaesthesia, we have shown the feasibility of detecting attempted movements during a state of ‘awake paralysis’. Patients might find themselves in such a state in various clinical situations, including settings on an intensive care unit. However, intraoperative awareness situations are generally not caused by a complete lack of general anaesthetics, but rather by a dose that is too low. Hence, the monitor should be able to detect (attempted) movement from the EEG even in the presence of low-dose anaesthetic drugs, which might influence the background EEG as well as the movement response (e.g. the latency, duration and kind of movement). Also the baseline state during sedation may be different from the instructed ‘no movement’ task in this study. To get a full understanding of the applicability of the paradigm during general anaesthesia, further studies are being conducted to determine the influence of those anaesthetics.

The presence of movement (attempt) alone might not be sufficient to conclude the patient is conscious, which represents another possible limitation^37^. Further research may be required to define the relationship between attempted and intended movement^38^.

Because of the burden of the study for the volunteers and the nature of the study — a feasibility test of a novel concept — the Medical Ethics Committee approved the study only for an absolute minimum number of participants. The number of four participants was found to be sufficient to show the strength of the brain response we aim to utilize, albeit with an expected broad confidence interval.

It is likely that the performance of the proposed system will improve when moving from the feasibility test presented here towards actual development of the BCI. This novel paradigm was evaluated in its simplest form: for a small participant group, single trial classification accuracies were determined using a basic classification algorithm. After this initial feasibility check, a number of steps can be taken to further improve the performance.

First, the amount of information the classifier uses for its decision could be increased. In this paper we reported the performance when a decision is made after only 6 seconds. Previously we have shown that performance increases when a longer movement period is used, with true positive response probabilities up to 100% within 2.5 minutes. Moreover, the system can be adjusted such that it can run for several hours without producing any false alarms^25^. To get an impression of the true positive/false positive tradeoff for attempted movement, we used a simple combination of detection threshold optimisation and combination of multiple classifier predictions for the current dataset, applied on the classification problem of training on actual movement and testing on attempted movement. An average false alarm rate of 0% with an average true positive rate of 87.5% was achieved when using approximately one minute of data.

Second, the movement task could be changed. For practical reasons of administering the neuromuscular blocker, the movement task in this study was limited to the right hand only. Because of the generally contralateral nature of ERD and ERS, the responses were especially strong in the left but not the right hemisphere of the brain. When moving both hands, the response will be elicited in a larger part of the brain, likely resulting in higher classification accuracies.

Third, the quality of EEG recordings in general is likely to increase. The current measurements took place in an operating room, with the full standard clinical setup. Conditions in such a location differ from the EEG rooms commonly used for BCI experiments. Although we acquired high quality signals, resulting in acceptable classification performance, we expect that future advancements in EEG hardware development will make BCI performance outside of the lab comparable to current performances acquired inside the lab within a few years.

Fourth, extended incorporation of sophisticated machine learning techniques should be investigated to further improve classification performance.

Concluding, despite its current limitations, the proposed paradigm has the potential to become a reliable addition or even an alternative to existing depth of anaesthesia monitors. We believe BCI technology in general and the attempted-movement-based monitor in particular are a promising new direction in the field of anaesthesia monitoring.

## Additional Information

**How to cite this article**: Blokland, Y. *et al.* Detection of attempted movement from the EEG during neuromuscular block: proof of principle study in awake volunteers. *Sci. Rep.* **5**, 12815; doi: 10.1038/srep12815 (2015).

## Supplementary Material

Supplementary Information

## Figures and Tables

**Table 1:** EMG power per condition as a percentage of EMG power during ‘actual movement’.

Subject #	actual movement	no movement	isometric	imagery	attempt
1	100	0.6	1.8	0.7	0.8
2	100	0.2	0.8	0.2	3.0
3	100	0.4	1.4	0.5	0.6
4	100	0.8	0.7	0.9	0.2

**Table 2:** Single trial cross-validated classification accuracies, expressed as percentages, for each movement condition and EEG channel set.

	30 EEG channels				9 EEG channels			
	Training & test conditions				Training & test conditions			
	Actual movement	Isometric movement	Imagined movement	Attempted movement	Actual movement	Isometric movement	Imagined movement	Attempted movement
Subject #								
1	81 (73–89)	83 (75–91)	73 (64–82)	78 (68–88)	78 (70–86)	80 (72–88)	69 (59–79)	81 (72–90)
2	83 (75–91)	68 (58–78)	55 (44–66)	73 (62–84)	81 (73–89)	68 (58–78)	56 (46–66)	82 (73–91)
3	93 (89–97)	89 (83–95)	73 (64–82)	92 (87–97)	96 (93–99)	88 (82–94)	78 (69–87)	92 (87–97)
4	79 (71–87)	81 (73–89)	75 (66–84)	81 (72–90)	92 (87–97)	77 (68–86)	80 (72–88)	79 (69–89)
Mean	84 (74–94)	80 (66–94)	69 (54–84)	81 (68–94)	87 (73–100)	78 (65–91)	71 (53–88)	84 (74–93)

First, separate classifiers were trained on each of the named movement conditions, using a subset of the recorded trials. Then the classification performances were estimated for each classifier on another set of trials belonging to that condition. For this procedure of performance estimation ten-fold cross-validation was used. Results are given as classification accuracy (95% CI): for each individual classification accuracy the *binomial* 95% CI is given, for the mean classification accuracy the standard 95% CI is given.

**Table 3:** Single trial classification accuracies for ‘attempted movement’, expressed in percentages, for each EEG channel set.

	30 EEG channels			9 EEG channels		
	Training conditions			Training conditions		
	Actual movement	Isometric movement	Imagined movement	Actual movement	Isometric movement	Imagined movement
Subject #						
1	71 (60–82)	70 (59–81)	68 (56-80)	71 (60–82)	66 (54–78)	59 (47–71)
2	76 (66–86)	81 (72–90)	67 (55–79)	69 (58–80)	83 (74–92)	58 (45–71)
3	89 (82–96)	84 (75–93)	67 (55–79)	91 (85–97)	90 (84–96)	73 (62–84)
4	80 (70–90)	70 (59–81)	73 (62–84)	78 (68–88)	70 (59–81)	77 (67–87)
Mean	79 (67–91)	76 (65–88)	69 (64–73)	77 (61–93)	77 (59–95)	67 (51–82)

First, separate classifiers were trained on each of the named movement conditions. Then, classification performances were estimated on ‘attempted movement’. Results are given as classification accuracy (95% CI): for each individual classification accuracy the *binomial* 95% CI is given, for the mean classification accuracy the standard 95% CI is given.
