# Supplementary material for: Detection of attempted movement from the EEG during neuromuscular block: proof of principle study in awake volunteers
Source: Sci Rep. 2015 Aug 7;5:12815. doi: 10.1038/srep12815 (PMC4528221; doi:10.1038/srep12815)
Supplement: Supplementary Information [file srep12815-s1.pdf]

Detection of attempted movement from the EEG during neuromuscular block: proof of principle study in awake volunteers

Yvonne Blokland, **Loukianos Spyrou**, **Jos Lerou**, **Jo Mourisse**, **Gert Jan Scheffer**, **Geert-Jan van Geffen**, **Jason Farquhar**, **Jörgen Bruhn**

Supplementary Figures

EEG time-frequency plots for channel C3 (top figures) and EMG power plots (bottom figures) per subject per condition. For each movement condition the top plot shows EEG power over time per frequency in relative units (r.u.). These plots were computed to ascertain the presence of event-related desynchronization (ERD, blue) and -synchronization (ERS, red), the main features the classifier uses for its decisions. A relative baseline over the entire trial was used, so that a value of 1 (white) represents average power, a value <1 a power decrease or ERD and a value >1 a power increase or ERS. The average EMG power over time is shown in the bottom plot. For the EMG plots a logarithmic scale is used on the y-axis.

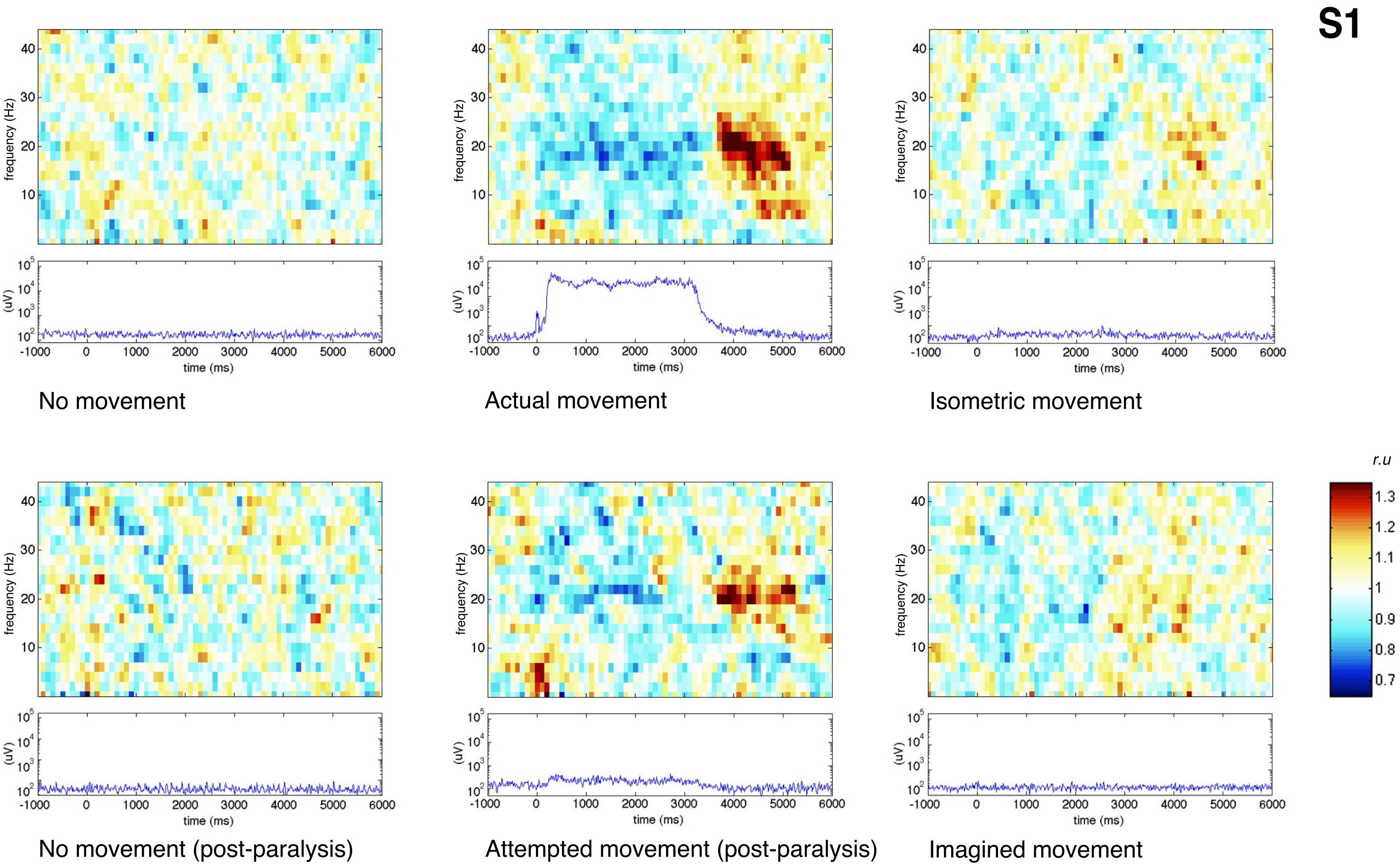
S1
frequency (Hz)
(uV)
time (ms)
No movement
Actual movement
Isometric movement
No movement (post-paralysis)
Attempted movement (post-paralysis)
Imagined movement
r.u

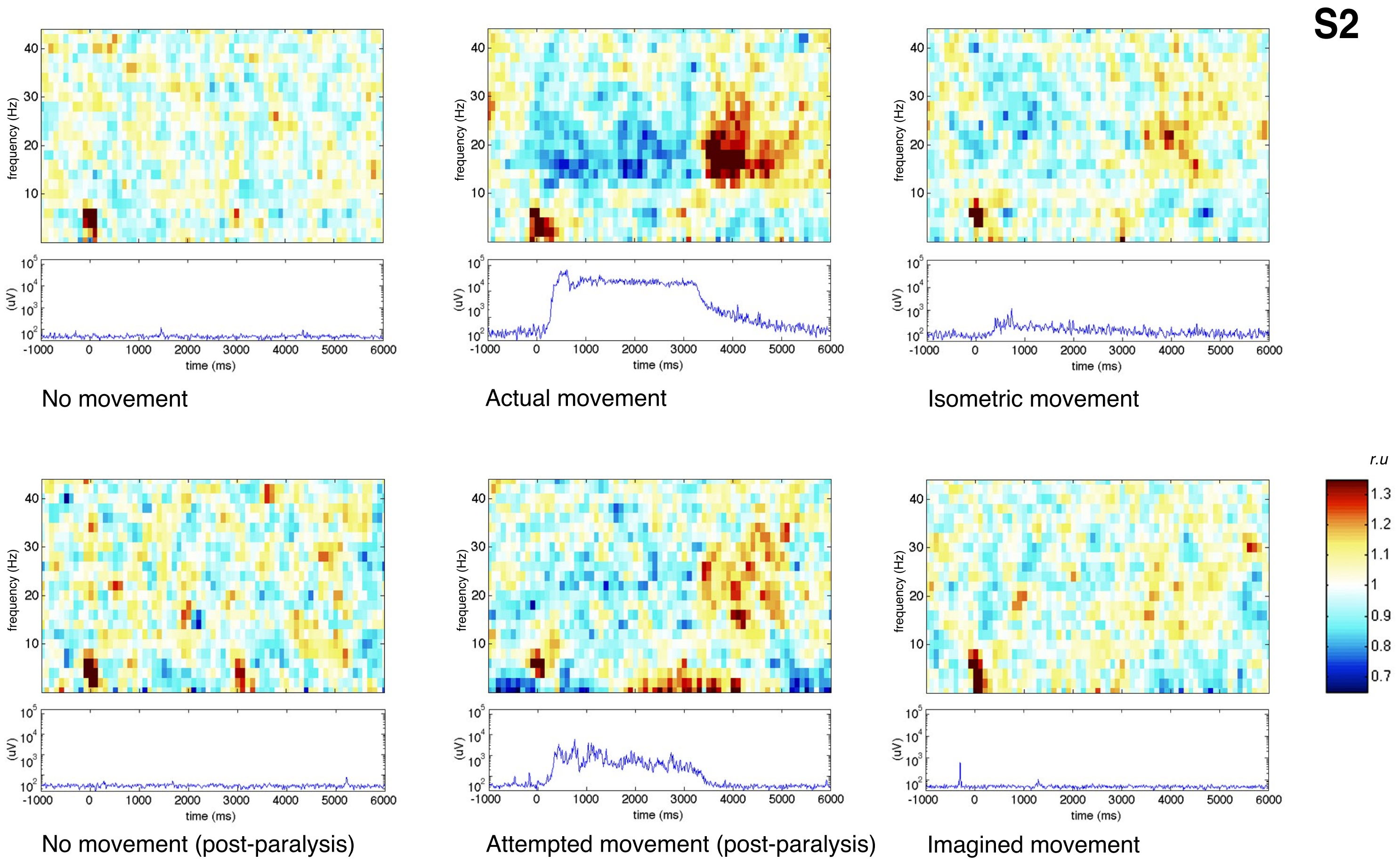
S2
frequency (Hz)
(uV)
time (ms)
r.u
No movement
Actual movement
Isometric movement
No movement (post-paralysis)
Attempted movement (post-paralysis)
Imagined movement

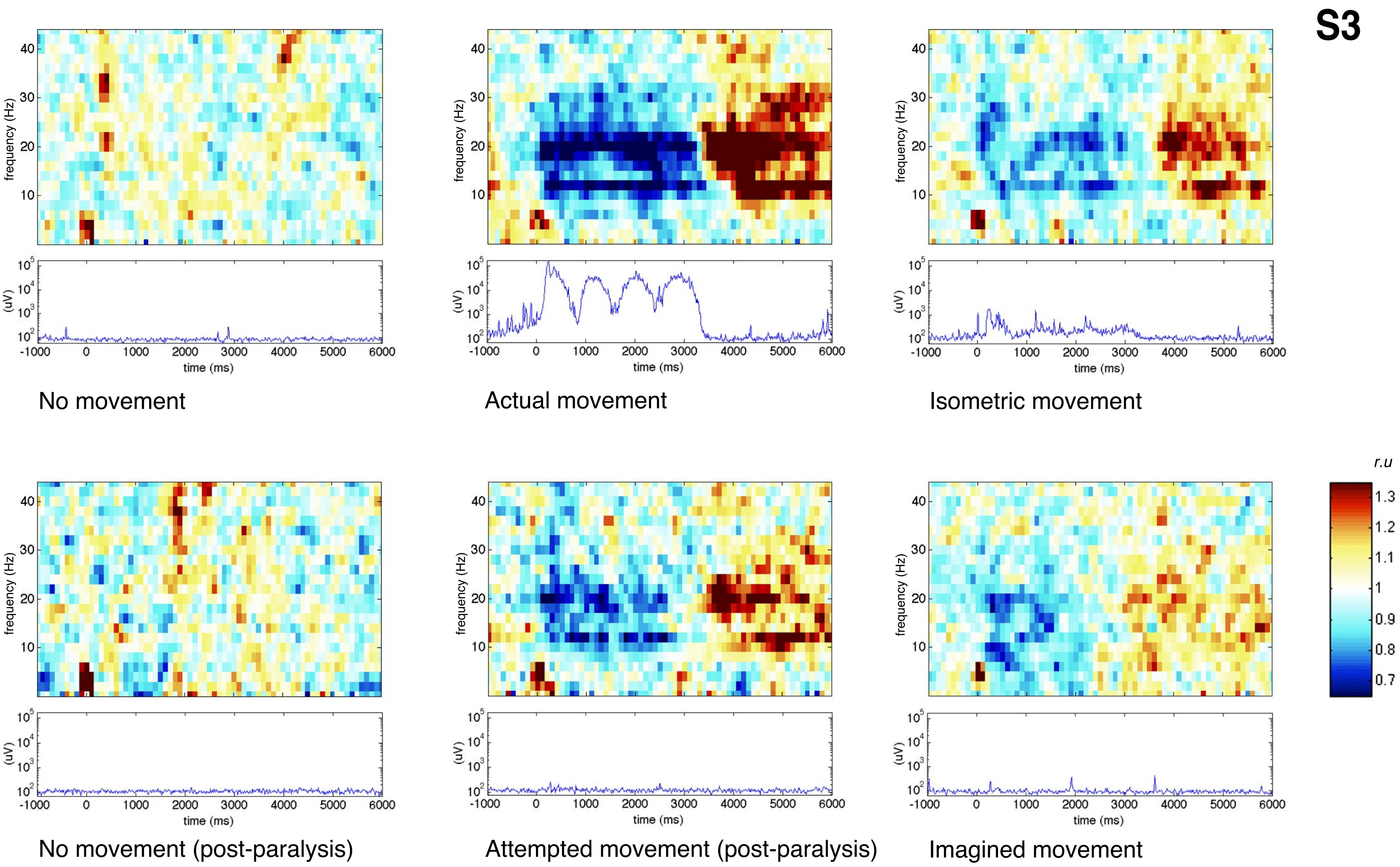
S3
frequency (Hz)
(uV)
time (ms)
No movement
Actual movement
Isometric movement
No movement (post-paralysis)
Attempted movement (post-paralysis)
Imagined movement
r.u
1.3
1.2
1.1
1
0.9
0.8
0.7

S4

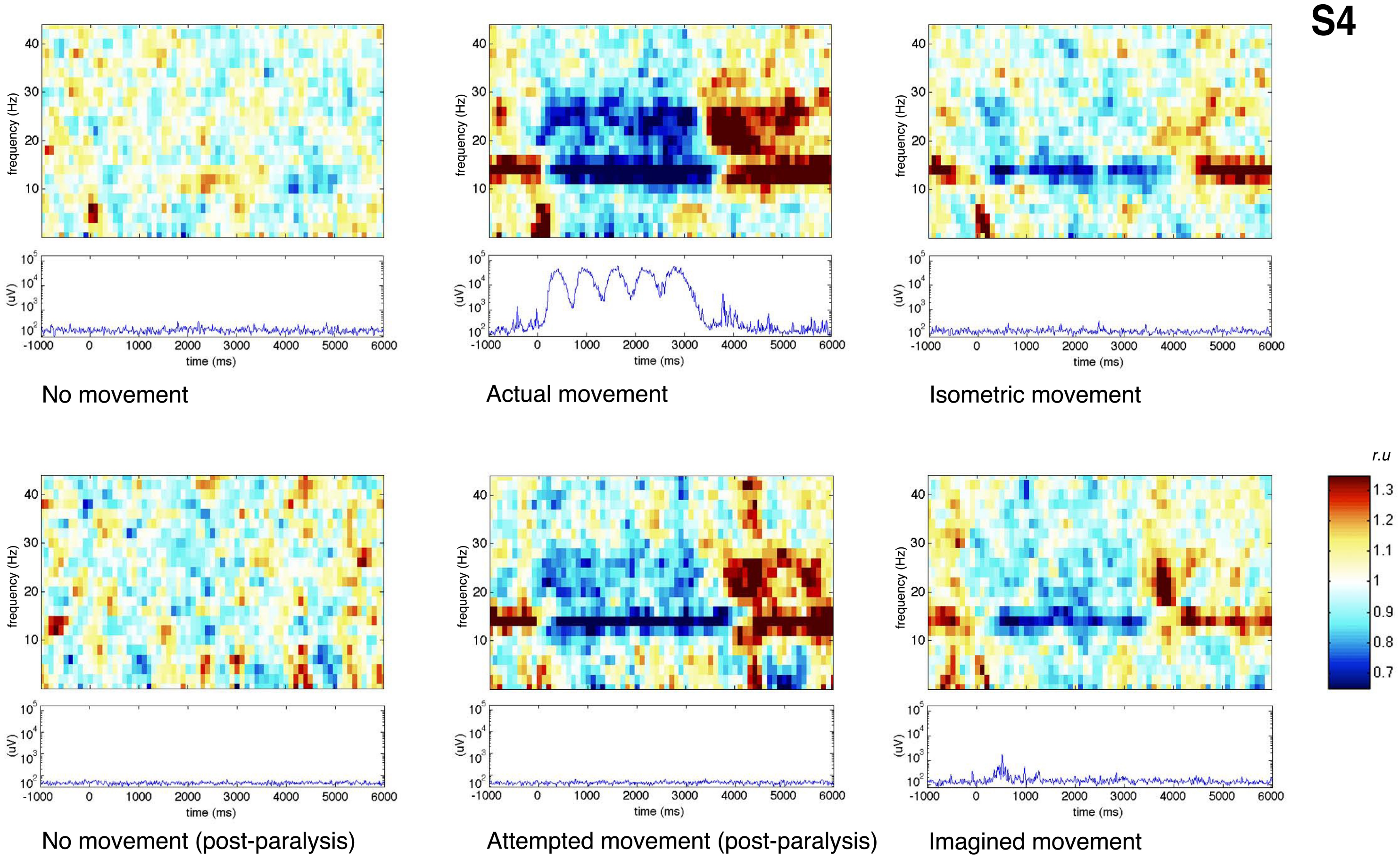
No movement
Actual movement
Isometric movement
No movement (post-paralysis)
Attempted movement (post-paralysis)
Imagined movement
frequency (Hz)
(uV)
time (ms)
r.u
